# Host species and pathogenicity effects in the evolution of the mitochondrial genomes of *Eimeria* species (Apicomplexa; Coccidia; Eimeriidae)

**DOI:** 10.1186/s40709-017-0070-2

**Published:** 2017-12-21

**Authors:** Asma Awadi

**Affiliations:** UR Génomique des Insectes Ravageurs des Cultures d’intérêt agronomique, Faculty of Sciences of Tunis, University of Tunis El Manar, 2092 Tunis, Tunisia

**Keywords:** *Eimeria*, Mitogenome, Selection, Host, Pathogenicity, Adaptation

## Abstract

**Background:**

Mitochondria are fundamental organelles responsible for cellular metabolism and energy production in eukaryotes via the oxidative phosphorylation pathway. Mitochondrial DNA is often used in population and species studies with the assumption of neutral evolution. However, evidence of positive selection in mitochondrial coding genes of various animal species has accumulated suggesting that amino acid changes in mtDNA might be adaptive. The functional and physiological implications of the inferred positively selected sites are usually unknown and are only evaluated based on available structural and functional models. Such studies are absent in unicellular organisms that show several crucial differences to the electron transport chain of animal mitochondria. In the present study, we explored *Eimeria* mitogenomes for positive selection. We also tested for association between mtDNA polymorphism and environmental variation (i.e. host species), parasite life cycle (i.e. sporulation period), and efficient host cell invasion (i.e. pathogenicity, prepatent period).

**Findings:**

We used site- and branch-site tests to estimate the extent of purifying and positive selection at each site and each lineage of several *Eimeria* parasite mitogenomes retrieved from GenBank. We founded sixteen codons in the three mtDNA-encoded proteins to be under positive selection compared to a strong purifying selection. Variation in the ratios of non-synonymous to synonymous changes of the studied parasites was associated with their different host species (F = 13.748; *p* < 0.001), whereas pathogenicity levels were associated with both synonymous and non-synonymous changes. This association was also confirmed by the multiple regression analysis.

**Conclusions:**

Our results suggest that host species and pathogenicity are important factors that might shape mitochondrial variation in *Eimeria* parasites. This supports the important role of mtDNA variations in the evolution and adaptation of these parasites.

## Findings

Mitochondria are important organelles for energy production and cellular signaling. These organelles are present in all eukaryotes where they show high variation in size and structure [1]. Positive and purifying selection has been extensively evaluated in mitochondrial DNA of a wide range of species, except for protozoans, showing that amino acid substitutions might suggest an environmental adaptation. Notably, protozoans were suggested to possess a different electron transport chain from those in animals with a respiratory chain composition that can vary depending on the growth conditions [2]. Parasites of the genus *Eimeria*, belonging to the phylum Apicomplexa, are responsible for coccidiosis, a disease of the intestinal tract in different domestic mammals and birds. The studied mitochondrial genomes of several *Eimeria* species were identical in structure and also in their genome organization [3–10]. These genomes, like other apicomplexan, are among the smallest ones in the eukaryotes to date and possess three genes encoding cytochrome c oxidase subunit I (COX1), cytochrome c oxidase subunit III (COX3) and cytochrome b (CytB), as well as numerous fragments of small subunits (SSU) and large subunit (LSU) rDNA [1, 4, 6].

Parasites of the genus *Eimeria* are useful organisms for testing hypothesis of selection on the mitochondrial genome due to their high genetic variability and their various host species. In this study, we tested for positive selection in the three encoding genes of several mitogenomes of the *Eimeria* parasite obtained from different host species. Provided positive selection on those mitogenomes and adaptation to different environments—mainly associated with the respective host species—we expected significant associations between non-synonymous variations and/or non-synonymous to synonymous ratios (dN/dS) with the host species. Moreover, as the ability to colonize successfully a host species depends in the pathogenicity and the prepatent period of the parasite, we also tested if these factors are associated with mitogenomes variability.

Twenty-five mitogenomes belonging to 19 species of the genus *Eimeria* retrieved from GenBank (Table 1) from earlier studies [3–10] were reanalyzed. We tested for positive and purifying selection in the mtDNA coding genes of *Eimeria* parasites. This aspect was not covered by any of the studies on mtDNA of this genus, where the mitochondrial data were only used to construct phylogenetic relationships.

**Table 1:** List of mtDNA genomes of *Eimeria* species downloaded from GenBank and used in the current study

GenBank accession numbers	Species	References
1. AB564272	*E. tenella*	Hikosaka et al. [3]
2. HQ702479	*E. acervulina*	Lin et al. [4]
3. HQ702480	*E. brunetti*	Lin et al. [4]
4. HQ702481	*E. maxima*	Lin et al. [4]
5. HQ702482	*E. necatrix*	Lin et al. [4]
6. HQ702483	*E. praecox*	Lin et al. [4]
7. HQ702484	*E. tenella*	Lin et al. [4]
8. JN864949	*E. mitis*	Lin et al. [4]
9. KC409029	*E. mitis*	Ogedenge et al. [6]
10. KC409030	*E. mitis*	Ogedenge et al. [6]
11. KC409031	*E. mitis*	Ogedenge et al. [6]
12. KF419217	*E. magna*	Tian et al. [8]
13. KF501573	*E. mitis*	Ogedenge et al. [7]
14. KJ608413	*E. gallopavonis*	Ogedenge et al. [7]
15. KJ608414	*E. meleagrimitis*	Ogedenge et al. [7]
16. KJ608415	*E. adenoeides*	Ogedenge et al. [7]
17. KJ608416	*E. dispersa*	Ogedenge et al. [7]
18. KJ608417	*E. gallopavonis*	Ogedenge et al. [7]
19. KJ608418	*E. meleagridis*	Ogedenge et al. [7]
20. KP009592	*E. intestinalis*	Liu et al. [9]
21. KP025690	*E. irresidua*	Liu et al. [9]
22. KP025691	*E. media*	Liu et al. [9]
23. KP025692	*E. vejdovskyi*	Liu et al. [9]
24. KP025693	*E. flavescens*	Liu et al. [9]
25. KR108296	*E. innocua*	Hafeez et al. [10]

Selection at specific amino acid positions was first assessed using the PAML 4 package [11] with the maximum likelihood method. Depending on ω values, i.e., the ratio of the rates of non-synonymous to synonymous nucleotide substitutions, sites were expected to evolve neutrally (ω = 1), under positive (ω > 1), or purifying (ω < 1) selection. Different codon-based models as suggested by [12, 13] were compared: scenarios where non-synonymous mutations were neutral or deleterious (M1 and M7, respectively) were compared with models that allowed for positive selection (M2, M3, M8); further details of our models used and the associated statistical background are given in [14]. Pairwise comparisons were performed using likelihood-ratio tests (LRT). Moreover, specific sites under selective constraints were also assessed using the Selecton 2.4 Server (http://selecton.tau.ac.il/) that estimates the Ka/Ks ratio on each codon [12, 15]. These ratios were then coded into colors that are projected onto the 3D structure of the protein. In the current study, M8 was used to estimate selection scores.

We further used additional five codon models implemented on the DATAMONKEY web server (http://www.datamonkey.org/) [16]. Single Likelihood Ancestral Counting (SLAC), Fixed Effects Likelihood (FEL), Random Effects Likelihood (REL), Fast Unconstrained Bayesian AppRoximation (FUBAR) and Mixed Effects Model of Evolution (MEME) [17, 18] were applied. Sites were considered to evolve under positive selection at significance level of *p* < 0.25 in SLAC and FEL [19] and *p* < 0.05 in MEME and Bayes factors > 50 in REL. In the above cited tests the best fitting model of evolution for each of the three coding genes, directly estimated on DATAMONKEY web server, was used.

Finally, we applied the branch-site test that target particular sites in specific branches that offer a strong tool to infer positive selection [20].

In order to assess the potential impact of the observed amino acid substitutions, HHPRED (http://toolkit.tuebingen.mpg.de/hhpred, last accessed March 10, 2016) [21] was used to remote protein homology for the three mitochondrial subunits. The three-dimensional (3D) structure of the closest homologs of each of the three coding genes was analyzed using the ConSurf webserver (http://consurf.tau.ac.il/, last accessed March 10, 2016) [22] in order to detect evolutionarily conserved and variable amino acids among all *Eimeria* mitochondrial coding genes. In the analysis, a specific score was obtained for each codon using the empirical Bayesian calculation method and mtREV model. These scores were distributed automatically into nine categories and assigned to different color codes according to the relative degree of conservation: most variable positions are classified into grade 1, whereas the most conserved ones are classified into category grade 9.

Evolution of parasites since the divergence from their ancestor might be shaped by environmental and host selective pressures. Therefore, we reconstructed the ancestral sequence of all *Eimeria* species using the maximum likelihood ancestral sequence reconstruction method implemented in the FASTML server (http://fastml.tau.ac.il/) [23]. We then calculated synonymous and non-synonymous changes and dN/dS ratios between the putative ancestral sequence and each sequence using DnaSP v5.1 [24]. We also calculated the overall number of positively selected sites in each sequence compared to its ancestor. Several factors might impact parasite evolution and these include host species, pathogenicity, prepatent period, and sporulation time. These characteristics were tested for association with the different amino acid changes parameters cited above. Minimum prepatent period, minimum sporulation time, and level of pathogenicity (low, moderate, high) were obtained from [25]. Corresponding hosts for the sequenced *Eimeria* species were obtained from the literature indicated in Table 1.

First, relationships between host species, pathogenicity and the different genetic parameters were assessed using an ANOVA test in SPSS^®^ vers. 18 (IBM, Chicago, USA). Then four multiple regression analysis were conducted using the genetic parameters as dependent variables and including host species, prepatent period and pathogenicity as independent variables. Notably, sporulation time was excluded from the analyses as it showed high correlation with the prepatent period as indicated by a Pearson correlation test.

**Fig. 1:**
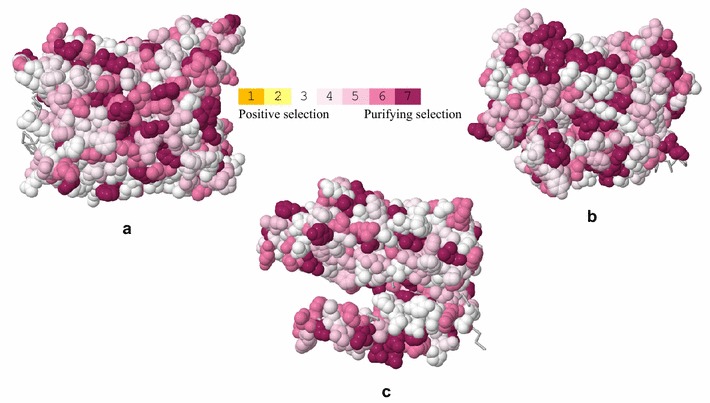
Selecton results for the three mitochondrial coding genes. The amino acids of the monomer are colored by their Ka/Ks scores using the color-coding bar. Significant purifying selected sites (*p* < 0.05) are colored in bordeaux (color number 7). **a** COX1, **b** COX3 and **c** CYTB

Comparisons of model results as obtained in PAML indicated the absence of sites under positive selection in *Eimeria* mtDNA (data not shown). Similarly, selection analysis on the Selecton webserver based on model M8 showed that all residues of the three coding genes were evolving either neutrally or under strong purifying selection (Fig. 1).

However, positive selection was suggested by the codon-based tests implemented in DATAMONKEY and the branch-site methods implemented in PAML package. All sites under positive selection as suggested by at least one of the applied tests are shown in Table 2. A total of 16 different codons in the three studied genes were under positive selection as suggested by REL, FEL, SLAC, MEME and FUBAR of which 6 sites were concordant in two of the used methods (Table 2). Four codons of the *E. mitis* and *E. brunetti* lineages were suggested to be under positive selection by the branch-codons analysis in PAML.

**Table 2:** Sites under positive selection according to six different methods in the three mitochondrial coding genes

	Site	Amino acid replacements	Aminoacid change					PAML
			SLAC	REL	FEL	MEME	FUBAR	Branch-sites
COX1	3	Y; S (2, 12, 14, 21, 22, 23, 24); P (15)	x	–	x	–	–	–
	177	T, A (19)	–	–	–	–	–	*E. mitis*
	199	G; L (2, 6, 7, 14, 15); V (1); A (5, 10)	–	–	–	x	x	–
	363	T; S (5, 9, 13, 25)	–	–	–	x	–	–
	365	V; L (2, 6, 7, 14, 18, 21, 23, 24)	–	–	x	x	–	–
	415	S; I (12)	–	x	–	–	–	–
	457	V; I (9, 12)	–	x	–	–	–	–
COX3	82	S; C (3, 17, 18, 19, 20, 21); A (16)	–	–	x	–	–	–
	135	I; V (17, 18, 19, 20)	–	–	–	–	–	*E. mitis*
	145	A; S (16, 22, 24, 25)	–	–	x	–	–	–
	208	T; V (2, 6, 7, 14, 24, 25), P (1, 5, 9, 10, 11, 13, 14); A (4, 8); L (12)	x	–	x	–	–	–
	218	S; N (3)	–	–	–	–	–	*E. brunetti*
CYTB	131	F, Y (12)	–	x	–	–	–	–
	185	F, Y (10)	–	x	–	–	–	–
	264	Y; N (10)	–	–	–	x	x	*E. mitis*
	347	V; L (9, 11, 13, 25); A (1, 12, 21); I (3); G (10), T (15)	x	–	x	–	x	–

List of amino acid replacements fixed within mtDNA lineages of *Eimeria* species (for species names see Table 1)

**Fig. 2:**
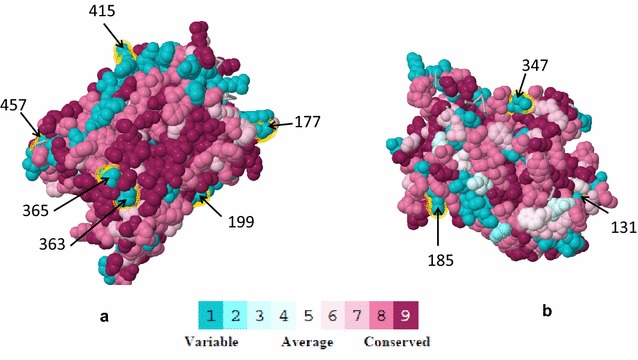
Topology and structure of the *B. taurus* superimposed with the multiple sequence alignment (MSA) of COX1 (**a**), COX3 (**b**), and CYTB (**c**) OXPHOS genes. The amino acids of the monomer are colored by their conservation grades using the color-coding bar, with turquoise through maroon, indicating variable through conserved amino acid positions. Amino acids under positive selection are also indicated

The impact of amino acid substitutions on the protein function was predicted for each subunit using the closest homolog protein structure. This analysis suggested higher conservation of genes of COX1 (HHPRED results: Prob = 100, Identities = 35%, Similarity = 0.629) and CytB (HHPRED results: Prob = 100, Identities = 44%, Similarity = 0.815) compared to COX3 (HHPRED results: Prob = 100, Identities = 22%, Similarity = 0.372) in comparison with the *Bos taurus* subunits. The Consurf analysis was possible only for COX1 and CytB where positively selected sites were indicated in the 3D structure (Fig. 2). The results suggested high variability, although functionally important regions seemed to be conserved on both the DNA and the protein at least for COX1 and CytB. However, the obtained low sequence identity did not allow assessing the impact of amino acid changes on the function of the analyzed mitochondrial coding genes. In this context, several studies have attempted to predict the impact of amino acid changes in the function and structure of mitochondrial proteins. However, most of those studies suggested that the effect of positively selected codons in various organisms seemed to be marginal as the mitochondrial coding genes are relatively conserved from mammals to bacteria [26].

Even in the absence of apparent functional changes, residues under positive selection were suggested to affect the efficiency of energy production in mtDNA. Such changes were interpreted, in animals, in the context of geographical and environmental characteristics such as high altitude [27, 28], temperature [29, 30] and food availability [28]. Moreover, analyses of adaptive evolution in 500 animal species [31] suggested that mitochondria undergo a significant amount of adaptive evolution, with an estimated 26% of non-synonymous substitutions fixed by adaptive evolution. The latter authors [31] suggested also that the rate of adaptive evolution was correlated to synonymous diversity, although the evidence was weak.

In the current study, we analyzed several parasite species infesting three different hosts. We found several codons under positive selection. Moreover, we found a significant effect of host species on dN/dS ratios as indicated by ANOVA test (Table 3). The same test showed also significant relationships between pathogenicity levels and synonymous and non-synonymous changes (Table 3). The effect of pathogenicity on synonymous and non-synonymous changes was confirmed by multiple regression analysis (Table 4). Notably, only synonymous changes were significantly affected by the three considered factors in our multiple regression model suggesting a combined effect of host species, pathogenicity levels and prepatent period on mtDNA substitutions (Table 4). The observed statistical association between the analysed phenotypes and mtDNA polymorphisms are supported by direct assays of mitochondrial mutants in animal infection models, and it can also be inferred indirectly from the requirement for mitochondrial function in cellular pathways associated with virulence (see [32] for an overview). Among others, the fact that mitochondrial function is necessary for virulence was suggested by a recent report of more virulent mitochondrial mutants of *Candida glabrata* [33]. Moreover, in *C. albicans*, inactivation of the putative subunits of the respiratory complex I resulted in attenuated virulence in the mouse model of systemic candidiasis [34, 35]. The attenuation of virulence has been suggested to be a result of a combination of reduced fitness, metabolic changes, and sensitivity to oxidative stress, which is linked to higher production of reactive oxygen species (ROS). In this context, it has been shown that some amino acid changes in mitochondrial subunits cause inefficiencies in the electron transfer chain system, contributing to the increase of ROS that can lead to the disruption of OXPHOS [32]. Ideally, the assessment of selection on various gene sequences coding for putative virulence factors or directly associated with the different phenotypes considered in the current study should complement our current results.

**Table 3:** ANOVA results: effects of Host species and pathogenicity on genetic parameters

Variable	Host	Pathogenicity
Synonymous changes	F = 1.186; *p* = 0.324	*F* *=* *4.016; p* *=* *0.033*
Non-synonymous changes	F = 2.159; *p* = 0.139	*F* *=* *3.641; p* *=* *0.043*
dN/dS	*F* *=* *13.748; p* *<* *0.001*	F = 2.801; *p* = 0.085
Positively selected sites	F = 2.922; *p* = 0.75	F = 2.375; *p* = 0.116

Significant values in italics

**Table 4:** Results of multiple regression analysis of each of the four genetic parameters and the three factors related to *Eimeria* parasites

Variable	Synonymous changes F = 3.297; *p* = 0.040			Non-synonymous changes F = 2.311; *p* = 0.106			dN/dS F = 2.244; *p* = 0.116			Positively selected sites F = 1.823; *p* = 0.174		
	β	t value	*p* value	β	t value	*p* value	β	t value	*p* value	β	t value	*p* value
Host	0.536	1.778	0.090	0.132	0.418	0.680	− 0.529	− 1.526	0.143	0.173	0.532	0.600
Pathogenicity	*− * *0.406*	*−* * 2.224*	*0.037*	*−* * 0.477*	*−* * 2.483*	*0.022*	− 0.410	− 2.081	0.051	− 0.398	− 2.019	0.056
Prepatent period	− 0.350	− 1.165	0.257	− 0.060	− 0.191	0.850	0.398	1.148	0.265	0.019	0.059	0.954

Significant association in italics

In spite of no immediate functional information on the presently found amino acid changes, our results show that the polymorphism in *Eimeria* mitochondrial coding genes might suggest an adaptation to host species. Indeed, the respiratory chain structure of these organisms varies greatly depending on external conditions [2]. Their electron transport chains are usually characterized by a lower efficiency of energy conservation when compared to the respiratory chains of animal mitochondria. Skulachev et al. [2] suggested that the main selective factor affecting the evolution of these electron transport chains is their ability to adapt to changing unfavorable external conditions. Indeed, in the unicellular eukaryote *Rhodotorula glutinis*, adaptation to deleterious environmental conditions (i.e. presence of aluminium) was attributed to mitochondria [31]. The *Eimeria* species are obligate intracellular protozoan parasites that experience a complex life cycle in the intestinal mucosa of the infected host [3]. These species are characterized by a single host life cycle and typically display a strict host and tissue specificity. For example, the chickens are parasitized by seven species of *Eimeria*, each restricted to a special part of the intestine [36]. Therefore, it is very likely that the host species is the major factor driving mitochondrial polymorphism. Indeed, these species have developed several adaptive characteristics to invade and survive within animal cells. Among these adaptive features, we can cite acquisition of adhesion protein domains and glycosylation systems through lateral transfer from animals (i.e. rhoptries and micronemes); diversification of the complement of apicomplexan surface proteins via massive lineage-specific expansions of certain protein families which are central to immune evasion; extensive protein polymorphisms and gene losses as a result of constant selective pressures from the host immune response [37]. Although little is known in this context, we expect that one key element in the successful invasion of the hosts is energy production by parasite species. Notably, apicomplexans utilize gliding motility that requires energy in the form of ATP [38] that is produced by oxidative phosphorylation in mitochondria. However, to confirm such hypothesis extensive analysis is needed. Structural protein mapping, testing protein activity, and analyzing fitness effect to test whether positively selected amino acids correspond to adaptive variations driven by environmental changes was suggested by [39].

In summary, we detected evidence of positive selection in several codons of the three mtDNA-encoded proteins, against a strong purifying selection in different *Eimeria* species. We also detected a significant association between host species, pathogenicity levels and amino acid changes. These results suggested that mtDNA changes might be adaptive, driven by a complex interactions between *Eimeria* parasite and their host species.
